# Supplementary material for: Quantifying Coexistence Concentrations in Multi-Component Phase-Separating Systems Using Analytical HPLC
Source: Biomolecules. 2022 Oct 14;12(10):1480. doi: 10.3390/biom12101480 (PMC9599810; doi:10.3390/biom12101480)
Supplement: Supplementary file 1 [file biomolecules-12-01480-s001.zip › biomolecules-1937236-supplementary_aep.pdf]

Supplementary Materials

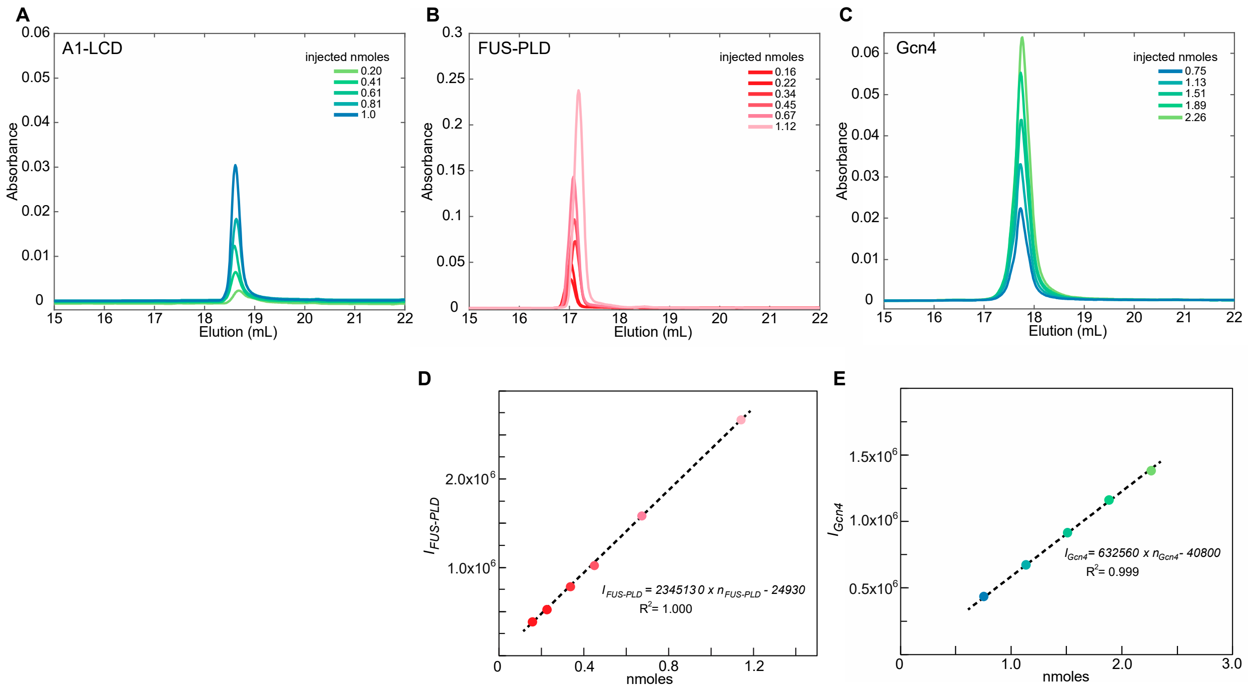

Figure S1. HPLC elution profile of **(A)** A1-LCD, **(B)** FUS-PLD and **(C)** Gcn4 were used to generate respective standard curves **(D)** and **(E)**, where the ordinate is the area under the HPLC elution peak, and the abscissa is *n*, given in nmoles. For the A1-LCD standard curve, see Fig. 2.

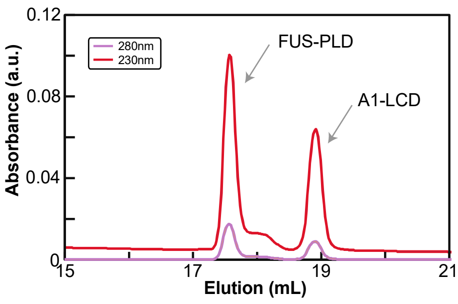

Figure S2. HPLC elution profile of A1-LCD and FUS-PLD monitored at 230 and 280 nm.
